# A nineteenth-century urban Ottoman population micro dataset: Data extraction and relational database curation from the 1840s pre-census Bursa population registers

**DOI:** 10.1038/s41597-024-03381-2

**Published:** 2024-06-03

**Authors:** M. Erdem Kabadayı, Efe Erünal

**Affiliations:** Koç University, Department of History, Istanbul, Turkey

**Keywords:** History, Sociology, Interdisciplinary studies

## Abstract

In recent decades, the “big microdata revolution” has transformed access to transcribed historical census data for social science research. However, the population records of the Ottoman Empire, spanning Southeastern Europe, Western Asia, and Northern Africa, remained inaccessible to the big microdata ecosystem due to their prolonged unavailability. This publication marks the inaugural release of complete population data for an Ottoman urban center, Bursa, derived from the 1839 population registers. The dataset presents originally non-tabulated register data in a tabular format integrated into a relational Microsoft Access database. Thus, we showcase the extensive and diverse data found in the Ottoman population registers, demonstrating a level of quality and sophistication akin to the censuses conducted worldwide in the nineteenth century. This valuable resource, whose potential has been massively underexploited, is now presented in an accessible format compatible with global microdata repositories. Our aim with this dataset is to enable historical demographic studies for the Ottoman realm and beyond, while also broadening access to the datasets constructed by our large research team.

## Background & Summary

We pursue two aims with this data publication. First and foremost, to facilitate historical demographic studies for the Ottoman realm and beyond. Secondly, making the datasets we constructed as a large research team accessible to a broader and hopefully growing audience. Between 2016 and 2022, the first European Research Council-funded research project on Ottoman demographic and economic history, ERC-StG, Grant Number 679097, “Urban Growth from the mid-nineteenth century Ottoman Empire to Contemporary Turkey in a Comparative Perspective, 1850–2000” with the acronym *UrbanOccupationsOETR*, was conducted at Koç University in Istanbul in collaboration first with the Cambridge Group for the History of Population and Social Structure, University of Cambridge and then the School of Geographical and Earth Sciences, University of Glasgow. M. Erdem Kabadayı was the principal investigator, and Efe Erünal was the longest-serving and lead researcher of *UrbanOccupationsOETR* (https://urbanoccupations.ku.edu.tr). We think the data contribution of our project is a timely addition to current developments in historical microdata.

The demographic dataset we provide (UrbanOccupationsOETR_1840s_Ottoman_Bursa_pop_micro_dataset, available via Zenodo)^1^ comprises the full population data of the city of Bursa recorded in the population registers of 1839 and updated until and including 1843. The population registers, taken in two waves in 1830 and 1839 and regularly updated, were part of the first empire-wide population enumerations of the Ottoman Empire and conducted contemporaneously with the censuses of other modern states. Although historical microdata of numerous countries is widely available in integrated public datasets and increasingly used in empirical studies, which has resulted in the “microdata revolution” of recent decades, the Ottoman population counts that enumerated millions of people have been unavailable for international research community going beyond the Ottoman studies. With this dataset, we present the originally non-tabulated register data in tabular format embedded in a relational database for the first time. In doing so, we demonstrate and explain the rich and varied data of the Ottoman population registers, whose quality and sophistication are on par with contemporary censuses conducted worldwide in the nineteenth century and whose potential has been massively underexploited, in an accessible format compatible with global microdata repositories.

The city of Bursa, a major cosmopolitan commercial hub in modern northwestern Turkey, is selected from the larger *UrbanOccupationsOETR* project database as an exemplary case to represent the volume, value, variety, and veracity of the population data, since urban areas are usually the most densely populated locations that attract the most migration in any country, they are attractive locations for multifold reasons in historical demography. As the project focused on urbanization and occupational structural change, it collected the population microdata on eight major urban centers (chosen as primary locations) and nineteen towns (denoted as secondary locations), which were of importance to the economic development of post-Ottoman nation-states (Table 1). *UrbanOccupationsOETR* focused on the mid-nineteenth-century Ottoman economic and demographic archival sources by prioritizing available population registers and taxation surveys. The geographical coverage of the mid-nineteen-century taxation registers is more limited than population registers^2^. Therefore, the availability of a relatively complete population and taxation register series played a decisive role in selecting eight regions and their urban locations. Bursa’s data is more advantageous than other cities because it has been cleaned and validated multiple times and used elsewhere for demographic and economic analyses (see below).

**Table 1:** A full breakdown of the regions (R) and their primary (P) and secondary (S) urban locations whose micro population data were extracted and entered into the UrbanOccupationsOETR project database.

GeoCode	Project Region	Province	District	Subdistrict	Urban Location	Household Count (HouseID)	Individual Count (IndivID)
R1_P	Ankara	Ankara	Ankara	Ankara	Ankara	4781	11142
R1_S1	Ankara	Ankara	Ankara	Ayaş	Ayaş	694	1984
R1_S2	Ankara	Ankara	Ankara	Murtazaabad	Murtazaabad	432	1342
R1_S3	Ankara	Ankara	Ankara	Nalluhan	Nalluhan	303	814
R2_P	Bursa	Bursa/Hüdavendigar	Bursa/Hüdavendigar	Bursa	Bursa	8391	19186
R2_S1	Bursa	Bursa/Hüdavendigar	Bursa/Hüdavendigar	Beypazarı	Beypazarı	911	2410
R2_S2	Bursa	Bursa/Hüdavendigar	Bursa/Hüdavendigar	Gemlik	Gemlik	479	1568
R2_S3	Bursa	Bursa/Hüdavendigar	Bursa/Hüdavendigar	İznik	İznik	170	383
R2_S4	Bursa	Bursa/Hüdavendigar	Bursa/Hüdavendigar	Mudanya	Mudanya	670	1650
R3_P	Plovdiv	Edirne	Plovdiv	Plovdiv	Plovdiv	2969	7817
R3_S1	Plovdiv	Edirne	Plovdiv	Plovdiv	Asenovgrad	1343	3143
R3_S2	Plovdiv	Edirne	Plovdiv	Plovdiv	Karlovo	1775	4813
R3_S3	Plovdiv	Edirne	Plovdiv	Plovdiv	Pazardzhik	2665	5287
R4_P	Ruse	Silistra	Silistra	Ruse	Ruse	4087	7644
R4_S1	Ruse	Silistra	Silistra	Ruse	Tutrakan	739	1393
R4_S2	Ruse	Silistra	Silistra	Razgrad	Razgrad	1210	3324
R4_S3	Ruse	Vidin	Vidin	Svishtov	Svishtov	1946	3798
R5_P	Bitola	Rumelia	Bitola	Bitola	Bitola	3543	10791
R5_S1	Bitola	Rumelia	Bitola	Bitola	Krushevo	1091	3499
R5_S2	Bitola	Rumelia	Bitola	Florina	Florina	769	1926
R5_S3	Bitola	Rumelia	Bitola	Prilep	Prilep	1565	5628
R6_P	Vranje	Skopje	Kyustendil	Vranje	Vranje	712	2015
R7_P	Thessaloniki	Thessaloniki	Thessaloniki	Thessaloniki	Thessaloniki	8607	17627
R8_P	Manisa	Aydın	Saruhan	Manisa	Manisa	5800	10725
R8_S1	Manisa	Aydın	Saruhan	Akhisar	Akhisar	952	1938
R8_S2	Manisa	Aydın	Saruhan	Menemen	Menemen	437	814
R8_S3	Manisa	Aydın	Saruhan	Turgutlu	Turgutlu	2990	7357
					**Total**	**60031**	**140018**

In what follows, we report on the recent explosion in the available historical microdata and the place of Ottoman censuses and census-like materials in it, then introduce the Ottoman population registers that were made available for research in the Ottoman archives in 2011, and finally, detail the process of tabulating the registers for Bursa and composition of the dataset^1^. We anticipate that the structure and content of this dataset will stimulate similar efforts in organizing large volumes of Ottoman social, economic, and demographic data spanning centuries and continents into accessible and interoperable relational databases and thus properly situate the Ottoman Empire in its much-desired place in the ever-expanding global historical microdata environment.

## The Global Microdata Revolution and the Place of Ottoman Population Data in it

In recent decades, a “big microdata revolution” has occurred in access to transcribed historical census data for social science research^3^. Besides the growing cooperation with national statistical offices, advancements in data entry, organization, preservation, harmonization, digitization, and dissemination technologies have exploded the volume of population microdata available worldwide^4^. Large-scale international collaborations such as IPUMS (Integrated Public Use Microdata Series)-International (https://ipums.org)^5^, Mosaic (https://censusmosaic.demog.berkeley.edu)^6^, and European Historical Population Samples Network (EHPS-Net) (https://ehps-net.eu), as well as genealogical organizations like Ancestry (https://www.ancestry.com), FamilySearch (https://www.familysearch.org), and Geneanet (https://geneanet.org), have enabled researchers to access billions of individual-level and interoperable tabulated population microdata from censuses, vital records, and census-like surveys extending over centuries from all over the world^7^. The versatility and spatial attributes of these microdata have significantly altered the fields of economic and demographic geography by allowing the study of various phenomena crucial to understanding the world we live in, such as the shift of labor force away from agriculture to industries and services, urbanization, and trends in birth, marriage, death, and migration, at granular geographic scales^8^.

Although polities across Eurasia throughout centuries partially succeeded in taking population counts at regional levels, the vast majority of the global repository of population microdata is from the last 200 years, when central governments across Eurasia and the Americas started to undertake consistent countrywide population enumerations in the late eighteenth century as a fundamental tool of state centralization and modernization projects. The initial censuses and census-like enumerations, such as those conducted in the United States in 1790, Netherlands in 1795, France, Denmark, and Great Britain in 1801, and Prussia in 1816, were tabulated headcounts/numeric with relatively little personal information such as ages, occupations, and marital status that aimed to determine mainly military, tax, and industrial potential and base administrative operations on a more empirical basis^9^.

The practice of nominative census-taking with standardized methods and definitions, developed by Adolphe Quetelet based on his census in the United Kingdom of Netherlands in 1829, was disseminated first to Great Britain in 1841, Belgium in 1846 (that Quetelet organized), the United States in 1850, and other countries in the subsequent decades, like the first censuses of Japan in 1872, British Colonial India in 1879, Mexico in 1895, and Russia in 1897. In these first modern censuses, *individual* replaced *household* as the registration unit, and many more variables, such as race and birthplace, started to be collected and updated with vital and migration statistics periodically by statistical bureaus. The sophistication of population data collection as a result of the development of statistical science served the dual aims of state-building and nation-building.

Spanning territories in Southeastern Europe, Western Asia, and Northern Africa, the Ottoman Empire started to enumerate its population in population registers in 1829, which recorded only males of all ages for conscription and taxation purposes, and implemented its first modern census, which included females, in 1881/1882, for more comprehensive statistical and governance reasons to converge with European census-taking practices and account for the increasing participation of females in economic and social spheres. Notwithstanding its wide expanse, estimated to have housed more than 39 million people within its 1893 borders^10^, the Ottoman Empire’s population records have not been a part of the big microdata ecosystem. This was, in large part, because of the long unavailability of these sources to researchers. Microdata of the population registers, which were conducted in 1830 and 1839, were made public by the Directorate of the Ottoman Archives only in 2011. The archives currently hold approximately 11,000 population registers, all accessible through the Directorate of the Ottoman Archives’ population registers catalog (NFS.d.) on their website (https://katalog.devletarsivleri.gov.tr) for full viewing and ordering to download in high-resolution JPEG format. The access to the rest of the empire-wide Ottoman censuses, namely the censuses of 1881/1882–1893 and 1906–1914, is restricted by the Turkish Ministry of the Interior, which keeps these records. Only per-location population totals exist for the last two censuses.

The microdata of late Ottoman censuses is only available for limited samples accessed through special ministry permission or special archives outside Turkey. The most prominent studies using Ottoman census material include Alan Duben and Cem Behar’s *Istanbul Households*^11^, written by taking five percent samples of five Muslim neighborhoods of Istanbul from the 1885 and 1907 censuses in the 1980s (data stored at Mosaic: http://www.censusmosaic.org/data/mosaic-data-files); the works of Johann Büssow^12^ and Michelle U. Campos^13^ on late Ottoman Jerusalem based on the censuses of the 1880s and early 1900s housed in the Israel State Archives; Daniel Ohanian, M. Erdem Kabadayı, and Mehmet Başkurt’s article^14^ on c. 1907 Armenian census of Istanbul based on the microfilm copies available at the Krikor and Clara Zohrab Information Center in New York City (data available at Houshamadyan: https://www.houshamadyan.org/mapottomanempire/vilayet-of-istanbul/istanbul/locale/demography.html); and Mohamed Saleh’s public release of the 1848 and 1868 population census samples of Egypt^15^, which was legally Ottoman territory until 1914 despite being a de facto independent state since the early nineteenth century (data published via IPUMS-International: https://international.ipums.org/international-action/sample_details/country/eg).

Another reason why the Ottoman population microdata accessible for over a decade has not been integrated into the international microdata repositories is that the majority of the existing literature using the population registers superficially utilized and failed to tabulate the microdata. Most works using these valuable sources contented with transcribing the microdata from Ottoman to Latin script and presenting their data in raw and unstyled fashion without publishing them in a separate repository. A considerable number of studies merely displayed population totals for locations with no spatial considerations. That said, a growing number (though still a minority and all belonging to the academic output of the initially mentioned *UrbanOccupationsOETR* project) of works have used the population registers critically, creatively, and geospatially for analyses of mortality^16^, migration^17,18^, agricultural land use^19^, and spatial^20^, ethnoreligious, and occupational distribution of populations^21^. Recent years have also witnessed the emergence of internationally funded large-scale projects that created high-quality geospatial datasets comprising per-location population data extracted from the population registers.

Among the large historical population projects are *UrbanOccupationsOETR* and *POPGEO_BG* (Marie Skłodowska- Curie Individual Fellowship, Population Geography of Bulgaria, 1500–1920: A Historical Spatial Analysis, PI: Grigor Boykov, https://popgeo.ku.edu.tr). They were funded by the European Commission and conducted at Koç University and geolocated all Ottoman settlements in central and western Anatolia and southeastern Europe^22^. Using around 850 population registers, these two joint projects entered toponyms and household and male population numbers into a geospatial dataset for a total of 18,502 settlements, of which 16,782 (91%) could be identified using satellite imagery and historical maps and geolocated using bespoke historical geographic information system (HGIS) applications. The resulting geospatial dataset containing over two and a half million males (about five million people in total) in almost a million households, whose first version was published in a mid-nineteenth-century gazetteer in 2022 (https://zenodo.org/records/11124537), is a key development in reconstructing the population geography of the Ottoman Empire. The provided mapping and the associated metadata are compatible with global historical geography datasets like *World Historical Gazetteer* (https://whgazetteer.org/public_data/) and *Gazetteer of British Place Names* (https://gazetteer.org.uk).

The combined efforts of *UrbanOccupationsOETR* and *POPGEO_BG* have documented the population and geographic information of the mid-nineteenth-century Ottoman population registers to a spatial extent and granularity never before achieved. Still, these registers’ coverage is much broader, and their data is much richer, which encourages geolocating all the populated places they cover and extracting all their information. The data of these registers have a wide variety of attributes that can transform the Ottoman economic and demographic history. But first, each attribute must be appropriately tabulated and managed to tap the full potential of and systematically use the registers for the whole registered settlements across the Ottoman Empire. A nuanced yet holistic and practical understanding of the population registers is required to optimally tabulate and manage various attributes belonging to millions of people.

As a pioneering work in making Ottoman population data in tabular format, the members of the *UrbanOccupationsOETR* team read, extracted, and entered the population data of certain urban centers and secondary towns/subdistrict centers in select regions of southeastern Europe and western and central Anatolia from handwritten Ottoman script into an integrated relational Microsoft Access database using customized data entry templates. The reason behind this data gathering is that one of the main objectives of the project was to analyze and compare the characteristics of urbanization and urban occupational structure across the Empire using the population registers in conjunction with the tax surveys of 1844–1845 (known as *temettuat*) which provide information on people’s estates and income sources. For these reasons, the urban locations of the imperial capital of Istanbul (massively overpopulated and tax-exempt) and the western Anatolian port city of Izmir, for which the registers and surveys could not be paired, were left out of the project’s scope. Although most of the urban locations were not included in our dataset, we still argue that having eight major urban centers with a further 19 urban locations in eight regions could be a representative whole of mid-nineteenth-century Ottoman urban demography. However, we must admit and stress that individual urban locations’ demographic characteristics vary considerably, and Bursa is a good example of not being representative of other urban centers. Stark residence pattern differences between mid-nineteenth-century Ankara, Bursa, and Thessaloniki have already been detected^23^. The lack of representativeness of Ottoman rural population datasets is even more evident. Due to the sheer share of the rural population and decisive local differences among populated places related to economic and social well-being, including climatic and environmental endowments, it is further difficult to look for demographic dynamics going beyond a few available examples. In relation to data quality, in our experience, rural population registers could still be reliable sources for total male population head counts due to their further use in updating demographic changes. They are also valuable and underutilized sources for labor migration^17,18^. However, due to the even more limiting impact of illiteracy in the countryside, the reliability of age data can be hypothetically lower.

A full breakdown of the regions and their primary (city/district capital) and secondary (town/subdistrict center) urban locations is provided in Table 1. A total of 60,031 households and 140,018 individuals were fully read and stored in the project’s population registers database. Note that the administrative units (province, district, and subdistrict) are as specified in the registers and that although a great majority of the households and individuals’ data date to 1839, the numbers in the table include the updates (i.e., newborns, migrants, and people who were absent during the initial registration period but subsequently added to the counts).

This data extraction and entry experience is important since urban centers and towns housed the largest concentration of people from different socio-economic and ethnoreligious backgrounds and witnessed occasional migration within and towards them. Therefore, their population data is replete with more varied qualitative and quantitative information and more frequently updated than that of rural settlements, which made up the great majority of the Ottoman population.

To exemplify the rich data of the population registers and how they can contribute to the global microdata revolution, this article presents a dataset of the 1839 population of the city of Bursa, a very populous, cosmopolitan, and one of the most occupationally diverse urban centers of the Ottoman Empire^1^. Not only is Bursa the largest settlement in our dataset, but its data is also the readiest for analysis, as will be outlined in the validation section below. But first, we concisely introduce the history, geographic extent, and contents of the Ottoman population registers, which is lacking in the literature.

## The Historical Context, Geographic Extent, and Contents of the Ottoman Population Registers

The Ottoman Empire carried out its first population registers in 1830 (AH. 1246), except for the capital Istanbul, where registration started in 1829 (AH. 1245). The set goal was to enumerate all male inhabitants in their households without exception. The registers’ first aim was to conscript able-bodied Muslims aged between 15 and 30 to the new regular army called the Trained Victorious Muslim Troops (*Muallem Asakir-i Mansure-i Muhammediye*), founded in 1826. Another aim was to determine the poll tax (jizya) eligibility of non-Muslim males (females and children were exempt). A third objective of the population counts was to provide a demographic basis for an individual and income-based tax system that would replace traditional collective taxation. The fourth objective was to track and, if needed, control the movement of persons within the Empire. To achieve these aims, the population registers were updated with birth, death, and migration information and with changes in military service and poll tax status.

The first registration period, whose updates continued until 1838, covered 14 out of the 29 provinces (*eyalet*) in total. The European provinces were Rumelia and Silistra (roughly limited to the area from modern Bulgaria, southern Serbia, and Montenegro in the north to Peloponnese in the south), and the rest corresponded to modern Turkey (minus provinces along today’s Syrian border), Mosul, Aegean islands, and Cyprus. The occupation of Egypt and the Levant between 1831 and 1840 by the rebellious governor of Egypt, Mehmed Ali Pasha, and the difficulty of conscripting Albanians, Bosnians, Kurds, Arabs, and nomads who had long enjoyed privileged autonomy limited the extent of registration^24^.

With the start of the administrative reform period called the *Tanzimat* in 1839, the second registration period started with more experience and better preparation. Although the 1839 registers had the same structure as in the previous period, they covered a larger area, now including Bosnia and Crete in the west and Syria, Lebanon, and Jerusalem in the east. The registers of the regions from southeastern Anatolia to Jerusalem began around 1844 (c. AH. 1260). European and Eastern Anatolian provinces (laying east of the Konya province, except the Black Sea coast) were recounted around this time, presumably to provide better estimates following the launch of the first conscription law and the first empire-wide estate and income (*temettuat*) surveys in 1844–1845. For this reason, the second wave of registers is mistakenly known in the literature as the “1844 census.” The registers were updated until the mid-1860s^25^.

The first– and second-wave population registers can be described as pre-censuses because they aimed to achieve specific political goals, recorded all but only males regardless of age, and did not tabulate their variables^26^. In terms of organization, they share the same methodology and structure. Instead of a comprehensive and clearly defined questionnaire, the Ottoman government issued general regulations regarding data collection, organization, and updates that were given to population officials who cooperated with local and religious headmen of local communities in keeping the master population register and updating them. The officials of local Population Bureaus (*Defter Nazırlığı*) in district centers were tasked to report several times a year military recruitment and vital events information, compiled based on notifications sent by local headmen, to the Population Registry Department (*Ceride Nezareti* - established along with local bureaus in 1831) in Istanbul, which preserved and updated the master registers. Population counts were annually compiled in summary (*icmal*) registers, which listed the total count of people and households by administrative division^27^.

The population registers of 1830 and 1839 classified the population under the commonly and officially recognized ethnoreligious identities - Muslim, Orthodox Christian, Armenian, Catholic, Jewish, and (Muslim and non-Muslim) Roma^28^. Muslim and non-Muslim populations were counted in separate registers. The registers were organized along spatial and temporal lines. The standard unit of the register was the quarter (*mahalle*) in urban and village (*karye*) in rural settings. Within these register units, populated public and non-household spaces such as inns, dervish lodges, monasteries, madrasas, coffeehouses, bakeries, mills, pastures (of nomads), and large private farms (*çiftlik*) were recorded separately.

**Fig. 1:**
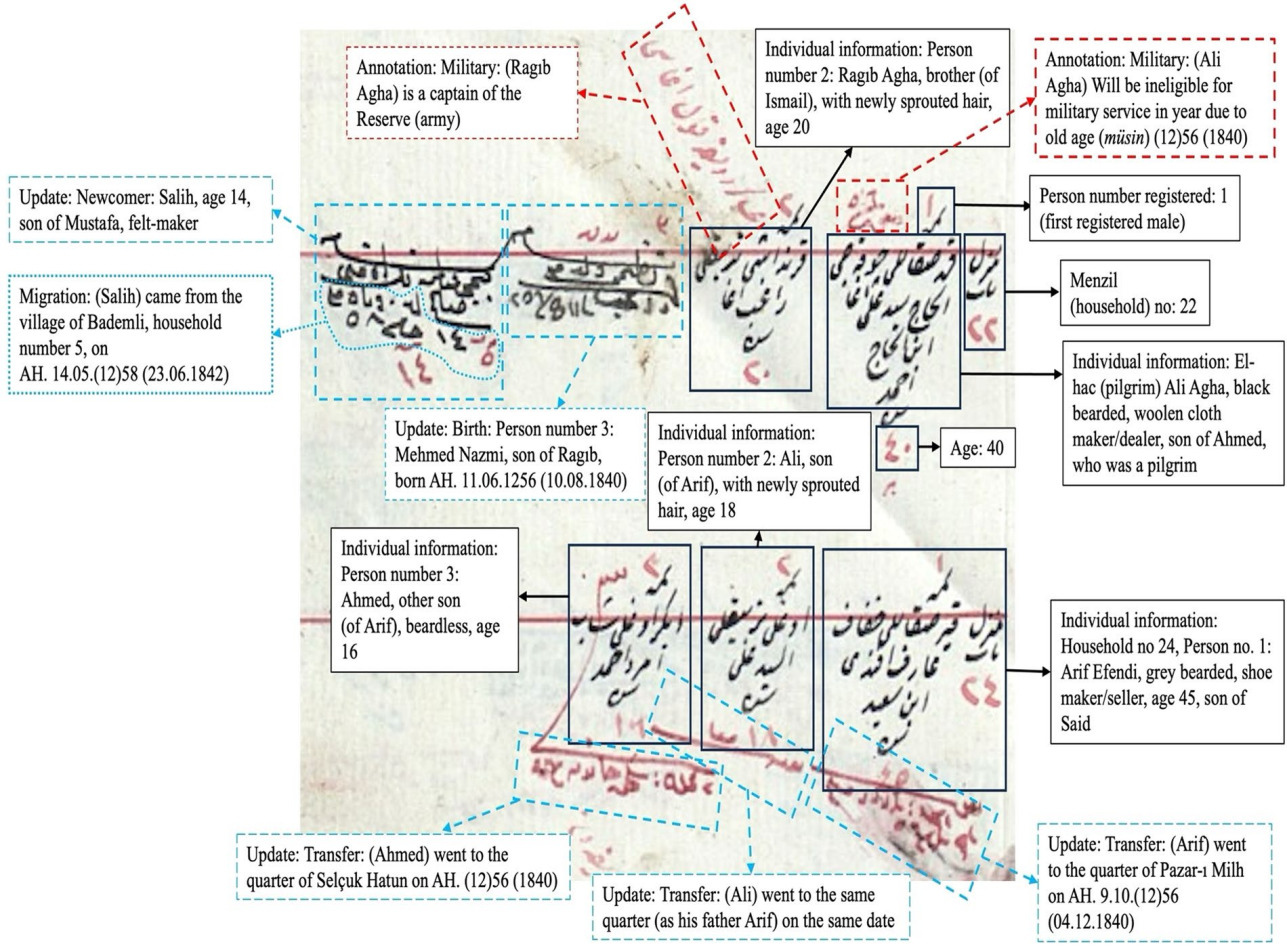
Snapshot from the Muslim register for Bursa, 1839 (NFS. d. 1396) (read from right to left).

The household (*menzil*/*hane*) was the unit of entry, which sometimes took different forms depending on the context, such as the room for inns and the tent for nomads. Each household recorded its members on a horizontal line (Fig. 1). The variables of male individuals inhabiting them were self-reported biographical information (names, titles/family names, ages, and occupations), physical description (height and facial hair), relationships with other household members (kinship, tenancy, and employment ties), infirmities, and military and poll tax status, including the reasons for exemption, military post, and poll tax category (high-*ala*, medium-*evsat*, and small-*edna*). Households without male members were sometimes identified by noting the name of the female head of the household, although full personal details were not provided within the household records. Furthermore, ownership information was documented for vacant households to ascertain the property owners. At the same time, if a resident was known to be absent during registration due to reasons such as military service or migration, he was recorded in his household with a note stating that reason. If he was missing and appeared later, he was added to the household during updates with a note like “not recorded previously” (e.g., *hin-i tahrirde taşrada olub*) or “newly recorded” (*tahrir-mande*).

In addition to the permanent residents of a given location, migrant/temporary non-local (*yabancı*) residents such as laborers, inn-stayers, and unemployed bachelors (*bî-kâr*) were recorded along with their place of origin and for how long they had been in the migrated place. Non-Muslim migrants were registered with information regarding the last location where they got their poll tax certificate and if they would make their next poll tax payment in the migrated location.

Updates were made mainly to births, deaths, migrations, and military and poll tax status. No other variables, such as age, were renewed except for occupations in a limited number of cases. Updates are easily identifiable since they were written in *siyakat*, a special Ottoman chancery shorthand script, and occasionally in red ink. Births were specified, and newborns’ names were added next to the father’s entry. Deaths were updated by crossing out the deceased person’s record. Migrations were added with a description of the migrated place (including the military branch if the person was conscripted). Military and poll tax status was updated by crossing out the old category and adding the new one next to it. Updates were usually expressed in hijri years, sometimes in month-year, and rarely in day-month-year fashion. It is important to note that since updates were made once every few months, these dates may reflect their registration date rather than giving the exact time of the events. Equally crucial is that many events, especially births, were not reported, so their quality is limited^16^.

## Methods

To showcase the varied content of the population registers, we present the population data of the city of Bursa collected in the second wave of registration in 1839. The population registers for the city of Bursa in 1839 are housed in the Directorate of the Ottoman Archives, listed in the population registers catalog under the archival codes NFS.d. 1396, 1398, and 7140. These registers are available for both viewing and ordering to download on the archives’ website, and they have been downloaded specifically for data entry purposes. They include the updates until and including 1843 (AH. 1259), whereas the rest of the updates are contained in separate registers. Muslims and Roma were recorded in NFS.d. 1396 and 7140, and non-Muslims (Orthodox Christians, Armenians, Catholics, and Jews) in 1398 (Figs. 1 and 5). Vital events registers were updated twice a year and recorded until 1864-65 (AH. 1280) and are accessible from the same catalog in the archives.

Bursa was the first capital of the Ottoman Empire in the fourteenth century and saw remarkable economic and demographic growth in the following centuries thanks to imperial investments in residential and public infrastructure promoting trade, its agriculturally fertile hinterland and proximity to Istanbul, and, above all, the city’s character as a raw silk and silk cloth production center and a major entrepot in the silk trade between Asia and Europe^25^. Although the establishment of mechanized silk filatures by European capitalists from the 1830s onwards negatively impacted the textile industries, Bursa continued to be an important administrative, industrial, and commercial city that attracted a relatively large number of migrants and one of the most populous urban locations in the Ottoman Empire and the Republic of Turkey.

Before presenting the demographic data, it is crucial to consider whether the city experienced significant population fluctuations. In the early nineteenth century, Bursa grappled with challenges like infections and fires due to its dense population. Despite events such as the 1801 fire and cholera outbreaks, widespread endemic diseases were absent, except for sporadic cases of malaria and dysentery. Effective quarantine measures in the 1820s helped control the spread of the plague, leading to its disappearance by the 1830s. This stability in population and health conditions makes Bursa in 1840 an ideal case study for demographic analysis, as it did not experience significant population shocks during the time of registration^16^.

**Fig. 2:**
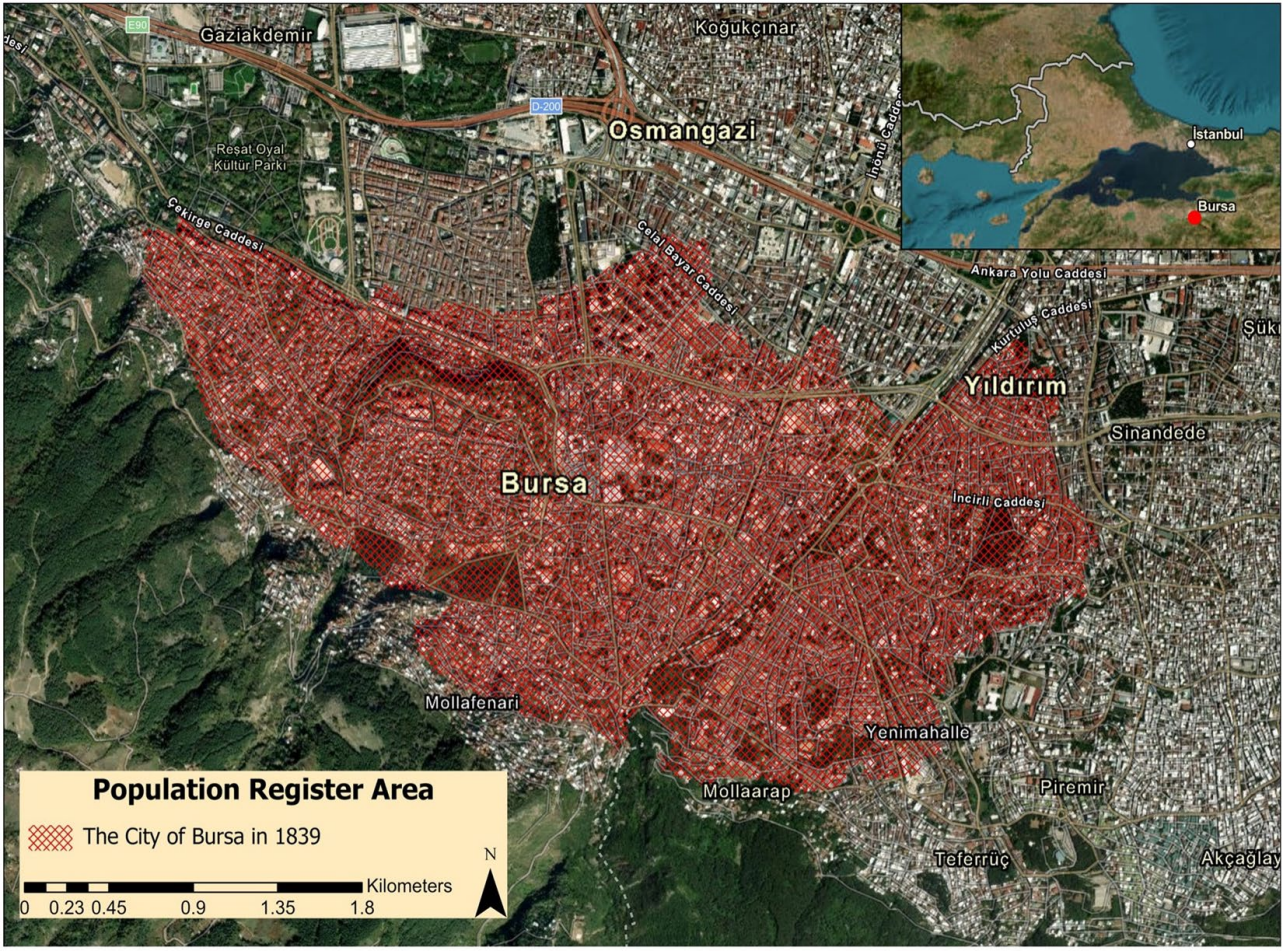
Area covered by the population registers for the city of Bursa, 1839, overlaid on current satellite imagery.

Given Bursa’s important place in Ottoman history and its relatively stable population, our dataset serves as a large and crucial resource for comprehending historical societal, economic, and demographic trends within the Empire in the early stages of globalization. The dataset has 8391 household entries (HouseID) and 19,186 individual (IndivID) entries^1^. This data includes the population registered in all of Bursa’s quarters and other residential categories (e.g., inns, bakeries, and coffeehouses) in 1839 and the updates until and including 1843 (Fig. 2). The ethno-religious breakdown of the total population is 12462 Muslims (65%), 3315 Armenians (17%), 2466 Orthodox Christians (13%), 749 Jews (4%), and 194 Catholics (1%).

## Cross-tabulation and Management of the Population Register Data for the City of Bursa

Converting non-tabulated population register variables into cross-tabulated format is essential to examining and making accessible the quantitative and qualitative information. Given that there are over a thousand available registers covering millions of households and individuals with many variables, powerful database software is required to enter, organize, manage, maintain, and analyze significant volumes of data. The *UrbanOccupationsOETR* project opted for Microsoft Access as its database system, which allows large data to be stored in related tables based on unique IDs. We are publishing the data first as a Microsoft Access database file because it is suitable for large datasets, its data is convertible to most data analysis software, and it can be easily manipulated with a simple query execution. To ensure that our dataset is more accessible, we are also releasing the dataset in Microsoft Excel format^1^.

**Fig. 3:**
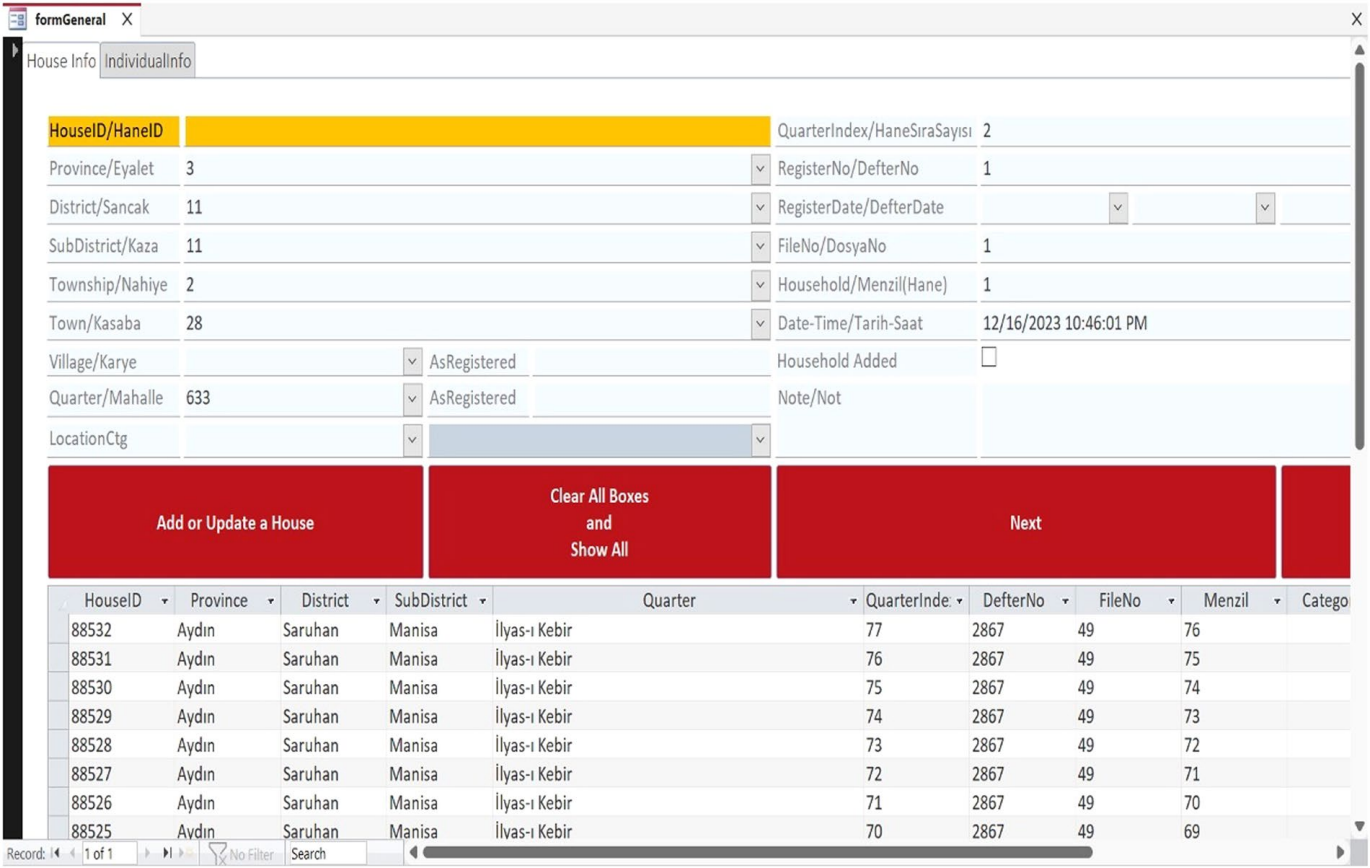
UrbanOccupationsOETR Microsoft Access data entry template, tab: HouseInfo.

**Fig. 4:**
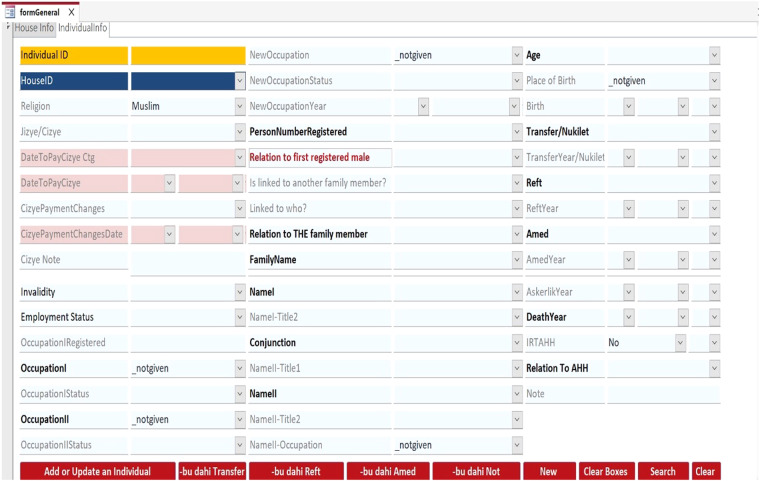
UrbanOccupationsOETR Microsoft Access data entry template, tab: IndividualInfo.

**Fig. 5:**
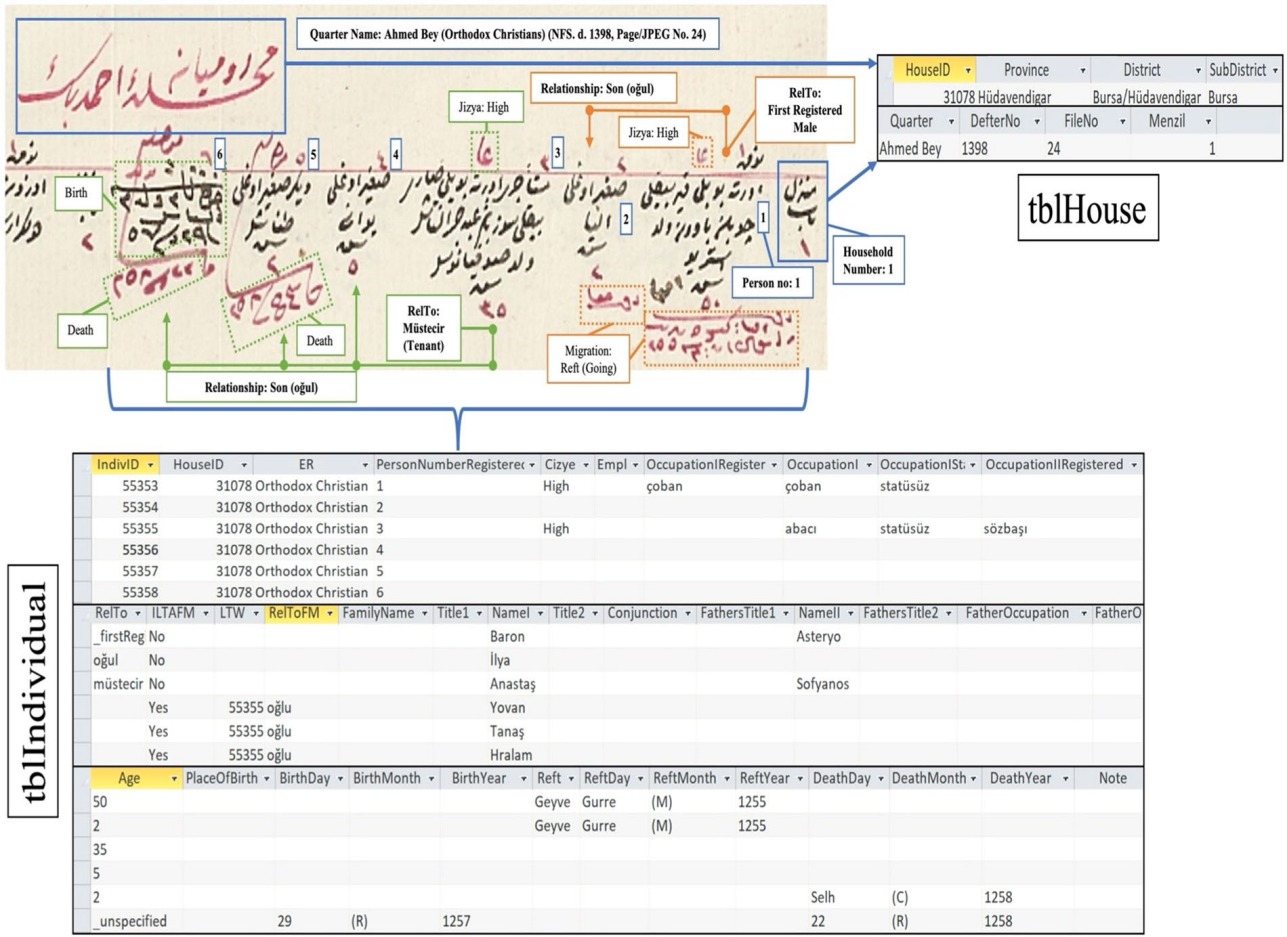
Snapshot from the non-Muslim register for Bursa, 1839 (NFS. d. 1398) (read from right to left), along with corresponding entries in tblHouse and tblIndividual.

The project team developed a custom data entry template to organize the population register data into a unified database (Figs. 3 and 4). Because the population registers’ data is structured in a hierarchy from geographic unit to household to individual, two tables were created, “tblHouse” for geographic units and households and “tblIndividual” for individuals. The project members extracted the data from the registers and manually entered the household and individual information using their respective data entry forms (Fig. 5). Households and individuals were automatically assigned a unique identifier when they were created. In terms of data relationships, a household identifier works as the primary key (“HouseID”) and links the tables together. “tblHouse” is the highest unit and has a “one-to-many” relationship to the individuals in “tblIndividual,” who have their unique identifier (“IndivID”) and a “one-to-one” relationship to the other household members.

## Data Records

**Fig. 6:**
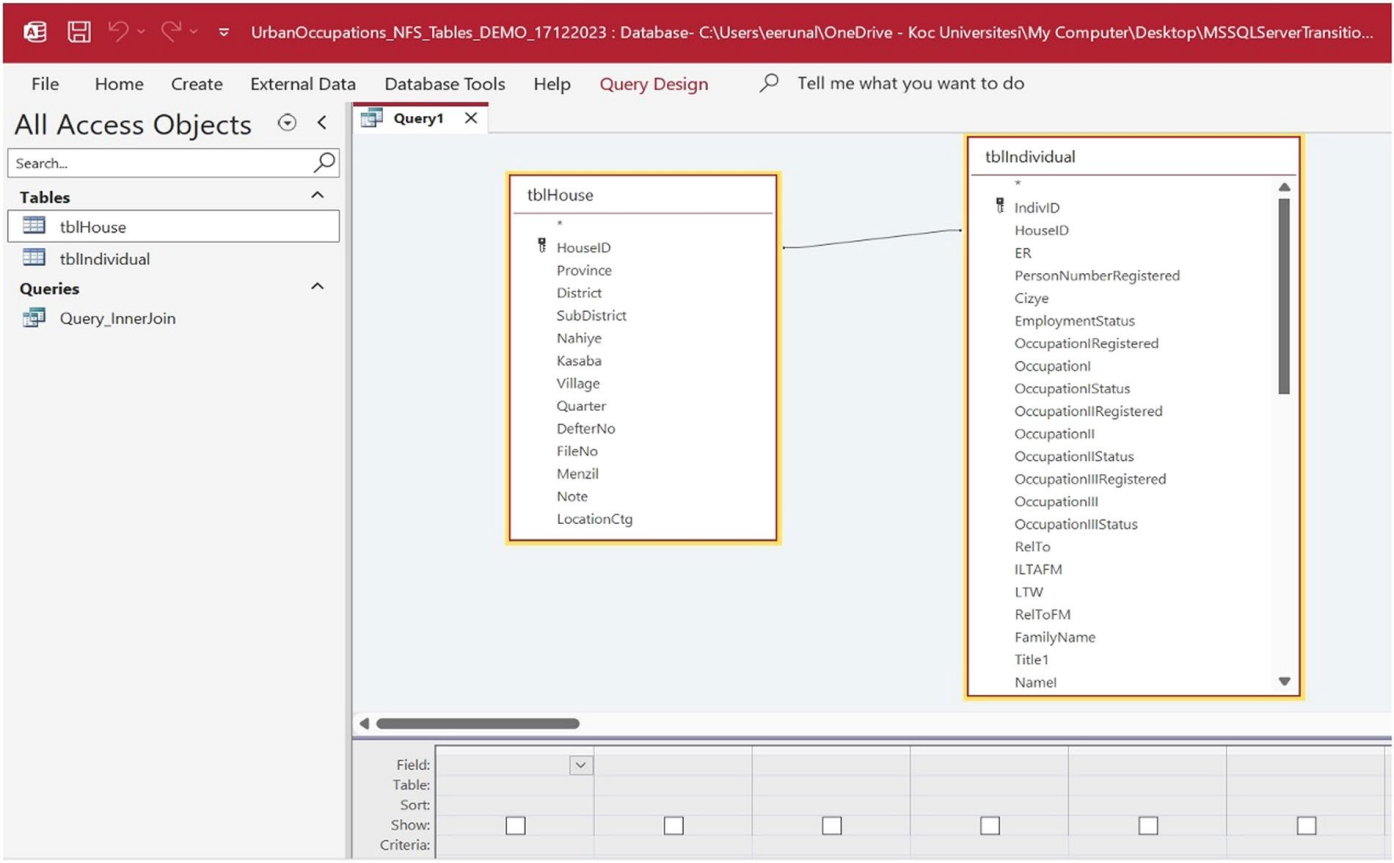
tblHouse and tblIndividual in Query Design view in UrbanOccupationsOETR Microsoft Access dataset.

UrbanOccupationsOETR_1840s_Ottoman_Bursa_pop_micro_dataset is published online via our project’s public dataset website (https://urbanoccupations.ku.edu.tr/public-datasets/) and Zenodo (https://zenodo.org/records/11124537), an open-access data repository developed and maintained by CERN under the European Open Science Infrastructure (OpenAIRE) program^1^. It presents a Microsoft Access database file (version 2016, 64-bit) containing the “tblHouse” and “tblIndividual.” Each table categorizes and standardizes the population register variables (Fig. 6). To make the data easier to use, the dataset also includes a query “Query_InnerJoin” that combines all the variables from each table in a separate sheet. Creating and sharing historical demographic data in this format and following our modalities will enable researchers to use both manual and query-based algorithmic record linkage, which will go beyond existing limitations in Ottoman historical demography. Furthermore, to make our data more accessible, we are also releasing all the tables in Microsoft Excel format. We hope our published dataset via this international and cross-disciplinary venue will make it accessible beyond the circles of Ottoman and historical demography studies and give the Ottoman Empire the place it deserves in the growing global population microdata ecosystem.

Before delving into data specifics, we must emphasize two aspects of the data entry procedure. First, the register data was transcribed in Turkish using modern Turkish spelling and punctuation to keep the nuances of the original source. That said, because the original register information is largely presented in a standardized fashion and grouped under detailed variables, the data can easily be translated into other languages and coded into specific coding schemes such as the PST (primary, secondary, and tertiary) system of classifying occupations developed by the Cambridge Group for the History of Population and Social Structure^29^.

Second, in a similar vein, we intentionally left out certain categories of personal information from our data extraction. These excluded categories are body height, skin complexion, eye color, and facial hair. These particular personal details were recorded in the registers for identification purposes. However, this information was not consistently provided, and when it was, it lacked sufficient standardization to make it interoperable. For instance, body height was often categorized simply as short, medium, or tall, and skin complexion was typically described as light, dark, or occasionally black in the case of enslaved black individuals. Eye color was rarely mentioned unless it was an uncommon color like blue. Facial hair information also suffered from the same lack of standardization and was not consistently provided for every registered individual. Therefore, especially considering the availability of age data, we decided to exclude facial hair as there was no necessity to use it as a proxy for age, such as white hair or black beard.

Turning to another aspect, addressing the implications of the exclusion of females from Ottoman population registers is crucial, as this omission inevitably skews demographic data, making it difficult to accurately analyze population structure, fertility rates, household composition, marriage systems, employment trends, migration patterns, and gender disparities. Females were only mentioned by name or gender, albeit infrequently, in households where there were no males present. For example, in the city of Bursa in 1839, 110 households in our dataset had an annotation pointing to this phenomenon but were later populated by males, as their updates indicate. Nevertheless, the comprehensive inclusion of all males, regardless of age or ethno-religious identity, in our dataset marks a promising beginning, considering the lack of similar detailed microdata previously available. The eventual opening of late nineteenth-century Ottoman censuses that include females will provide the most realistic correction coefficients to enhance estimates for the mid-nineteenth century.

As a last note of caution, we should stress the fact that Ottoman population registers are not systematic or standardized records. They continuously evolved from their emergence in the 1830s until their replacement with the first universal population censuses in the 1880s. The recording practices became more inclusive from year to year and in selected locations, responding to the administrative needs of the Ottoman central state, such as conscription or sedentarization. They started to register non-Muslim and/or non-resident populations more rigorously due to taxation purposes. In this respect, for a chosen location and period, if there are multiple available series of registers, one should compare the data quality. We opted for this strategy when constructing our dataset.

To broaden access and use of our data and bring it more in line with findability, accessibility, interoperability, and reusability (FAIR) data guidelines, the variables of each table are sorted into general categories and described in detail (see Supplementary Table 1). As the variables indicate, the dataset and population registers, in general, could serve to formulate unprecedented, hitherto impossible research questions related to major demographic dynamics, e.g., household size and composition, ethnoreligious differences, population density, occupational structure, intergenerational mobility and status transfer, mortality, fertility, migration, age-heaping/human capital, conscription, settlement patterns within and across urban locations, onomastics, toponymy, etc.

Furthermore, specifically related to the Ottoman studies, we also hope that this dataset will serve as a blueprint for organizing and homogenizing the rich and hitherto underutilized Ottoman demographic and socioeconomic data – be it the household-based land surveys (*tahrir*) of the fifteenth, sixteenth, and the seventeenth centuries, the Ottoman tax surveys of 1844-1845, or the late Ottoman censuses.

## Technical Validation

Ottoman population registers have been made available to research relatively recently, and there has hitherto been no attempt to tabulate and make their data accessible. A critical outlook has yet to emerge in the slowly accumulating literature using the registers. Therefore, validating the register data with heterogeneous variables published for wide reuse for the first time is challenging. However, we are confident that the urban Bursa population dataset we release is the readiest for analysis within the *UrbanOccupationsOETR* project database^1^.

More than any other location in the *UrbanOccupationsOETR* database, UrbanOccupationsOETR_1840s_Ottoman_Bursa_pop_micro_dataset underwent several post-processing steps by multiple team members who controlled the data from beginning to end, ensured data accuracy and reliability by taking random samples and checking the variables against the original register information, and cleaned the data of errors. Still, even if all data is entered correctly and without error, the veracity of the register information can only be tested by cross-checking it with other contemporaneous evidence.

Several studies using the population registers of Bursa city in juxtaposition with other independent historical accounts and data have evaluated the quality of the register information and the dataset. In his PhD thesis, Efe Erünal manually matched individuals in the city of Bursa’s population registers in the 1844-1845 tax surveys, post-mortem probate inventories (*tereke*), and tombstones to create economic and demographic biographies of people and households^25^. This original micro-level data matching experiment has shown a very high level of consistency of individual information across all the sources that are years apart. Erünal’s thesis also employed the population register’s data and annual summary totals to conduct a long-term population density analysis of the entire district of Bursa/Hüdavendigar, including the city of Bursa, the subdistrict centers, and rural locations. In doing so, the thesis geolocated 591 populated places in the district and 166 neighborhoods of the city and found that the population of every settlement was enumerated without exception and regardless of ethno-religious identity. The thesis also found that population density patterns are consistent with the population statistics of the later and better Ottoman censuses as well as with contemporary population estimates of the foreign consuls residing in Bursa. Population increase trajectories per subdistrict, on the other hand, are in harmony with the number of migrants coming to the district largely as a result of wars with Russia.

In an article published in 2021, Erünal used the population register of the city of Bursa to conduct mortality rate and age distribution analyses for an entire urban location for the first time in Ottoman history^16^. The age distribution of the city’s stable (only permanent) population corresponds to Coale and Demeny’s Regional Model Life Tables (East, GRR = 3.00, L = 4-6) that were based on theoretical stable populations undisturbed by migrations, compiled from the registration data of actual populations worldwide, and are the most popular model life tables among demographers. Nevertheless, Erünal’s attempt to examine infant and child mortality revealed that the birth and death updates are imperfect, especially for children, due to high death rates among them and to the seasonality of vital registration and update periods. However, when mortality rates are calibrated according to Coale and Demeny’s table that best fits Bursa’s population in 1839, the infant mortality rate (IMR) of between 336.5 and 393.6 (meaning that 3 to 4 in every 10 children dying before their first birthday) closely follows the IMR experience of the contemporaneous Central and Eastern European countries and regions where birth and death data quality was more frequently collected. Regarding the age structure, Erünal’s study found that the age data for the city is significantly affected by age-heaping to the nearest five-year interval. The tendency of the informants to heap their ages suggests that the data of the registers actually provide self-reported information (in other words, not through the mediation of some other people or institution). It is also in line with the large body of literature using the prevalence of age-heaping as an indicator of numerical skills and a proxy to measure human capital, which is relatively low in countries with little economic growth and very limited access to formal education like the Ottoman Empire.

M. Erdem Kabadayı used the 1839 population registers belonging to the cities of Bursa, Ankara, and Thessaloniki together with their tax surveys from 1844-1845 to compare their occupational structure using the PST system of classifying occupations, with a particular emphasis on people employed in the public sector, since each source covers a large proportion of the working males in the cities^23^. Kabadayı’s study mapped occupational structures onto residential patterns and found that the sources covered the majority of the neighborhoods, with Bursa achieving full coverage. The fact that each city had its unique occupational structures shaped by regional economic developments, resource endowments, and geographical location further proves the authenticity of the self-reported biographical information in the registers.

Finally, Yekta Said Can, a computer scientist and an affiliated researcher of *UrbanOccupationsOETR*, in two articles co-authored with M. Erdem Kabadayı, validated the age data in the dataset for Nicaea (İznik), a historical and industrial town northeast of the city of Bursa, in the district of Hüdavendigar, whose population data was read and extracted by the same team responsible for entering the Bursa city’s data. Nicaea was chosen as a pilot study region because its registers are clean and loosely placed due to relatively little population and updates. Can and Kabadayı trained a CNN (Convolutional Neural Network) model for carrying out automatic page segmentation and automatically spotting individual entries^30^ and the Arabic numerals^31^ in the population registers themselves. Then, Can and Kabadayı assisted this model with an image processing algorithm to detect objects (individuals and residential unit markers) in the registers and with the Deep Transfer Learning method from large open Arabic handwritten digit datasets for digit recognition. Their results achieved 100% accuracy in spotting individuals and correctly assigning them to their residential units (households and quarters) and above 99% recognition accuracy for handwritten digits. Not only do these results increase confidence in the usefulness of the project datasets, but they also prove promising for feeding the Ottoman population and economic data to machine reading algorithms and retrieving their information in bulk in time and energy-saving ways.

### Supplementary information

Supplementary Table 1: Categories and descriptions of the variables of tblHouse and tblIndividual

## Data Availability

No custom code was employed in the research presented in this publication. *UrbanOccupationsOETR*_1840s_Ottoman_Bursa_pop_micro_dataset is freely available via *UrbanOccupationsOETR* public dataset website (https://urbanoccupations.ku.edu.tr/public-datasets/) and Zenodo (https://zenodo.org/records/11124537), and no password is required for download or utilization.
