# Supplementary material for: A nineteenth-century urban Ottoman population micro dataset: Data extraction and relational database curation from the 1840s pre-census Bursa population registers
Source: Sci Data. 2024 Jun 3;11:570. doi: 10.1038/s41597-024-03381-2 (PMC11148089; doi:10.1038/s41597-024-03381-2)
Supplement: Supplementary file 1 — Supplementary Table 1: Categories and descriptions of the variables of tblHouse and tblIndividual [file 41597_2024_3381_MOESM1_ESM.pdf]

Categories and descriptions of the variables of tblHouse and tblIndividual

**tblHouse**		
**Category**	**Variable**	**Description**
**Unique key/ID**	"HouseID"	Unique and consecutive ID belonging to a specific household, automatically generated by Microsoft Access
**Geographic unit of entry**	"Province" & "District" & "SubDistrict" & "Village" & "Quarter"	Geographic unit of entry from province to quarter as it appears in the register
**Register specifics**	"DefterNo"	Archival code of the register whose data is being entered
	"FileNo"	JPEG number of the register page of the household being entered
	"Menzil"	Number of the household (specified by the registers as *Menzil*, Persian word for house), as appears in the register
	"Note"	When a household is annotated with a note, e.g. it is empty (e.g., *zükurdan ve inasdan hali, nüfusdan hali*), populated only by females (e.g., *zükurdan hali, nisvan tasarrufunda*), or in different use (such as a dervish convent)
	"LocationCtg"	Specifies if the household refers to a dwelling other than a household, like an inn or a madrasa

tblIndividual		
**Category**	**Variable**	**Description**
**Unique key/ID**	"HouseID"	Unique ID belonging to a specific household, automatically generated by Microsoft Access, which links the individuals to households and connects the "tblHouse" and "tblIndividual"
	"IndivID"	Unique ID belonging to a specific individual, automatically generated by Microsoft Access
**Ethno-religious identity**	“ER”	Ethnoreligious identity of the individual as given in the register
**Tax information**	“Cizye” (Poll tax / jizya)	Poll tax category of the individual when it was first recorded
	“DateToPayCizyeDay” & “DateToPayCizyeMonth” & “DateToPayCizyeYear” & “DateToPayCizyeCtg”	When the individual will be eligible for poll tax in hijri dates and the tax category to be paid
**Occupation**	“EmploymentStatus”	Specific references to one’s non-working status such as retirement [*tekaüd*, *amelmande*]
	“OccupationIRegistered”	Occupation as registered in the record [e.g., barber’s apprentice in someone’s shop]

	“OccupationI”	Standardized version of the occupation [e.g.,barber]
	“OccupationIStatus”	Status of the occupation [e.g., apprentice]. If an occupation did not have a status, then “statüsüz” (no status) was entered.
	“OccupationIIRegistered” & “OccupationII” & “OccupationIIStatus” & “OccupationIIIRegistered” & “OccupationIII” & “OccupationIIIStatus”	Versions of the descriptors above if the individual is employed in multiple occupations
**Individual interrelationships**	“RelTo”	Short for “Related To,” and shows the individual’s relationship or lack thereof to the first recorded male within the household. If he was the “_firstRegisteredMale,” then he is recorded as such. The type of relationship to the “_firstRegisteredMale”, such as son (*oğlu*), grandson (*hafidi*/*torunu*), tenant (*kiracı*), or slave (*gulamı*/*kölesi*), or lack of it (in the case of a new individual moved to the household) are specified.

	“ILTAFM”	Short for “Is Linked to Another Family Member.” When the individual is related to someone other than the “_firstRegisteredMale,” only “Yes” or “No” is chosen here
	“LTW”	Short for “Linked to Whom.” If the “ILTAFM” is “Yes,” "LTW" specifies the “IndivID” of the household member an individual is related to
	“RelToFM”	Short for “Relationship to the Family Member,” and shows the relationship of the person to the “LTW”
**Individual data**	“PersonNumberRegistered”	Number assigned to an individual that is automatically generated and can be manipulated by the data entry person if needed (e.g., if the number does not match the one in the register)
	“FamilyName”	The individual's family name, in the rare occasion before the official introduction of family names (not to be confused with title)
	“Title1”	Title(s) appearing before an individual’s name

	“NameI”	The individual's name(s)
	"Title2”	Title(s) appearing after an individual’s name
	“Conjunction”	Arabic patronymic *ibn, bin*, and *veled* (equivalent to the “-son” suffix in English, meaning the son of "NameII") and Turkish patronymic *oğlu* (the opposite, meaning the son of "NameI", appearing rarely), that links "NameI" and "NameII"
	“FathersTitle1"	Title(s) appearing before the father’s name (if the conjunction is "oğlu", then the title of the son (the individual himself)
	“NameII”	The father's name(s) (if the conjunction is "*oğlu*", then the name of the son (the individual himself)
	“FathersTitle2”	Title(s) appearing after the father’s name (if the conjunction is "*oğlu*", then the title of the son (the individual himself)
	“FatherOccupation”	The father’s occupational information
	“FatherOccupationStatus”	The father’s occupational status

	“Age”	Ages expressed in years. If the ages are given in months or days for under-one age children in the register, they are converted to years and shown in fractional form (e.g. 6/12 for a six-months-old child and 2/354 for two-days-old baby)
	“PlaceOfBirth”	The individual’s birthplace or place of origin
	“Note”	If the individual is annotated with a note, or a data entry person needs to highlight an issue regarding an individual’s data, they are entered to this column
**Birth Updates**	“BirthDay” & “BirthMonth” & “BirthYear”	A newborn’s birth date as expressed in hijri dates, in the available detail
**Death Updates**	“DeathDay” & “DeathMonth” & “DeathYear”	The individual’s death date as expressed in hijri dates
**Migration Updates**	“Transfer”	Specified by the registers as *nakil* (Arabic word for "transfer") and refers to the movement destination within a location, like from one household to another

	"TransferDay" & "TransferMonth" & "TransferYear"	The individual's "Transfer" date as expressed in hijri dates
	"Reft"	Destination of emigration (specified by the registers as *reft,* Persian word for "going")
	"ReftDay" & "ReftMonth" & "ReftYear"	The individual's "Reft" date as expressed in hijri dates
	"Amed"	Where an individual came from (specified by the registers as *amed* - Persian word for "coming"). If an individual is exempted from military service and returned home, this information was entered here.
	"AmedDay" & "AmedMonth" & "AmedYear"	The individual's "Amed" date as expressed in hijri dates
		Note: If there were multiple migration events for an individual, multiple "Transfer," "Reft," and Amed" data and their dates were separated by a slash ("/").
**Military updates**	"Askerlik" (Turkish word for military service)	The individual's military status, including eligibility (recorded as *tuvana*, meaning strong, and shortened as "t" when presented along with

		other information), and/or reasons for exemption e.g., old age (recorded as *müsin*, meaning aged, and shortened as “m” when presented along with other information), infirmities (e.g, *yekçeşm* meaning one-eyed), and retirement (*tekaüd*, *mütekaid*)
	“AskerlikDay” & “AskerlikMonth” & “AskerlikYear”	Specifies data such as when an individual’s service started, when an eligible (“t”) male was to be conscripted, and when a male was exempted or would be exempt (“m”) as expressed in hijri dates
		Note: If there were multiple military data for an individual, they are given together (e.g. "yekçeşm m" [one-eyed and (m)üsin/exempt]).
**Special day, month, and year abbreviations**	“Evail” & “Evasıt” & “Evahir” & “Gurre” & “Selh”	Hijri dates are sometimes shown with special abbreviations. For days, “Evail” refers to the first ten days of a month; “Evasıt” to the middle ten days and “Evahir” to the last ten days of a month. “Gurre(-i)” is used for the first day of a hijri month and “Selh(-i)” is for the last day.

	(M) & (S) & (RA) & (CA.) & (C) & (B) & (Ş) & (N) & (L) & (ZA) & (Z)	Months can be shown in Latin letters referring to the hijri month: Muharrem: (M), Safer: (S), Rebiül-evvel: (RA), Rebiül-ahir: (R) Cemaziyel-evvel: (CA), Cemaziyel-ahir: (C), Receb: (B), Şaban: (Ş), Ramazan: (N), Şevval: (L), Zilkade: (ZA), Zilhicce: (Z).
	Two and three-digit years	Years are sometimes expressed in two or three digits instead of four by ignoring the millennium or the century when the registers were completed. In this case, for example, year “58” should be read as 1258 and “259” as 1259
**Special terms**	“_undeciphered”	If a variable could not be read, "_undeciphered" was entered
	“_unspecified”	If a variable was not given, such as the age of the newborn, "_unspecified" was entered
	“2000”	If one part of a variable could not be read, then "2000" was entered in its place (e.g., "2000oğlu" [“2000son”] in place of a family name where

		only "oğlu" part is legible)
	"kz" & "nh" & “ks” & "kr" & "h" & "mdrs" & "m"	In "Birthplace," "Reft," "Amed," and "Transfer," the following codes were used if they were specified in these sections in the registers: "kz" or *kaza* (subdistrict), "nh" for *nahiye* (township), “ks” for *kasaba* (little town/borough), "kr" for *karye* (village), "h" for *han* (inn), "mdrs" for madrasa, and "m" for *mahalle* (quarter). If these variables could not be read, then "_undeciphered_" was entered before these codes, and if they were not specified, then "_unspecified_" was entered before them (e.g., “_undeciphered_kz”)
